# Kyasanur Forest Disease Outbreak and Vaccination Strategy, Shimoga District, India, 2013–2014

**DOI:** 10.3201/eid2101.141227

**Published:** 2015-01

**Authors:** S.K. Kiran, Achhelal Pasi, Satish Kumar, Gudadappa S. Kasabi, Prabhakara Gujjarappa, Aakash Shrivastava, Sanjay Mehendale, L.S. Chauhan, Kayla F. Laserson, Manoj Murhekar

**Affiliations:** National Institute of Epidemiology, Chennai, India (S.K. Kiran, S. Mehendale, M. Murhekar);; National Centre for Disease Control, Delhi, India (A. Pasi, S. Kumar, A. Shrivastava, L.S. Chauhan, K.F. Laserson);; Department of Health and Family Welfare, Shimoga, India (G.S. Kasabi);; Thirthahalli Taluk Hospital, Thirthahalli, India (P. Gujjarappa);

**Keywords:** Kyasanur Forest disease, outbreak, vaccination, India, viruses, vector-borne diseases, zoonotic diseases

## Abstract

We investigated a Kyasanur Forest disease outbreak in Karnataka, India during December 2013–April 2014. Surveillance and retrospective study indicated low vaccine coverage, low vaccine effectiveness, and spread of disease to areas beyond those selected for vaccination and to age groups not targeted for vaccination. To control disease, vaccination strategies need to be reviewed.

In India, Kyasanur Forest disease (KFD), a tickborne viral hemorrhagic fever that occurs as seasonal outbreaks during January–June (*1**,**2*), has been endemic to 5 districts of Karnataka State. However, during 2012–2013, KFD infection was reported from other districts and states in India: Chamarajanagara District, Karnataka State; Nilgiri District, Tamil Nadu State; and Waynad District, Kerala State (*3*).

Vaccination with formalin-inactivated tissue-culture vaccine has been the primary strategy for controlling KFD. The strategy involves mass vaccination in areas reporting KFD activity (i.e., laboratory evidence of KFD virus [KFDV] in monkeys, humans, or ticks) and in villages within a 5-km radius of such areas (Directorate of Health and Family Welfare Services, Government of Karnataka, 2005 manual on Kyasanur Forest disease; unpub. data). Two vaccine doses are administered at least 1 month apart to persons 7–65 years of age. Vaccine-induced immunity is short-lived, so the first booster dose of vaccine is recommended within 6–9 months after primary vaccination; thereafter, annual booster doses are recommended for 5 years after the last confirmed case in the area (*4*).

Beginning in January 2014, increased cases of unexplained fevers were reported from Thirthahalli Taluk, a subdistrict of Shimoga District (Figure 1). On February 6, 2014, the National Institute of Virology (Pune, India) confirmed the presence of KFDV in 5/12 serum samples from patients. We investigated the outbreak to describe the epidemiologic characteristics of KFD, estimate vaccine effectiveness (VE) and coverage, and propose recommendations for control.

**Figure 1:**
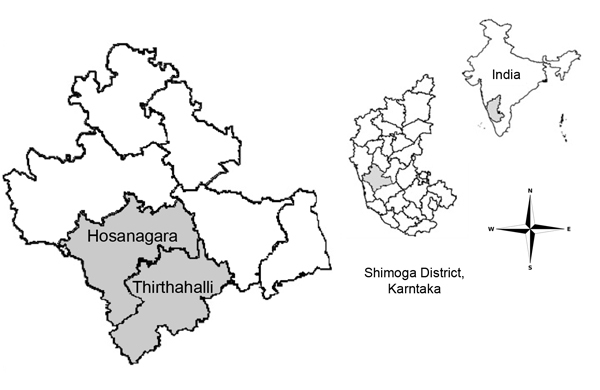
Map of Shimoga District, Karnataka State, India, showing the location of Thirthahalli and Hosanagara Taluks, which were affected by an outbreak of Kyasanur Forest disease virus during December 31, 2013–April 7, 2014, and other taluks within the state. The smaller inset maps show, respectively, the location of Shimoga District within Karnataka State and the location of Karnataka State within India.

## The Study

We established KFD surveillance in 3 large public health facilities in Thirthahalli Taluk: Thirthahalli Taluk Hospital, Kannangi Community Health Center, and Konandur Primary Health Center. Patients from neighboring Hosanagara Taluk also seek care at these facilities. A suspected case was defined as sudden onset of fever, headache, and myalgia in patients attending these facilities during the last week of December 2013 through the first week of April 2014. Medical officers at surveillance facilities collected information regarding each patient’s age, sex, place of residence, and clinical and vaccination history. Serum samples from all suspected case-patients were tested for KFDV at the National Institute of Virology by using reverse transcription PCR (RT-PCR) (*5*). RT-PCR–negative samples were tested by ELISA for KFDV IgM (*5*). Samples were also tested for dengue virus and *Leptospira* spp. We analyzed the data to describe the disease by time, location, and person. We used the population of affected villages and applied the age- and sex-distributions of Karnataka State’s population (2011 census) to the population of the affected villages to calculate attack rates.

To calculate vaccine coverage, we obtained 2013 KFD vaccination data from district health officials. To estimate VE, we conducted a retrospective cohort study in 4 villages within the Kannangi Community Health Center catchment area: Garaga-Kikkeri (466 residents in 106 households), where KFD vaccination was conducted in 2013, and the neighboring villages of Kannangi, Kombinakai, and Avalagere (total of 528 residents in 146 households), where vaccination was not conducted. We systematically sampled 60 households from the vaccinated village and 110 from the nonvaccinated villages and collected information from persons 8–66 years of age about the number of KFD vaccine doses received in 2013. Information about laboratory-confirmed cases in vaccinated and unvaccinated persons was obtained from surveillance data. We estimated the relative risk (RR) of acquiring KFD associated with vaccination and then calculated VE as follows: VE = 1 − RR.

During December 31, 2013–April 7, 2014, facility-based surveillance identified 246 suspected cases of KFD; 106 (43.1%) patients were positive for KFDV (78 by RT-PCR, 28 by IgM ELISA); 1 case-patient was also positive for dengue-specific IgM. The laboratory results of suspected case-patients screened up to March 2014 are available elsewhere (*6*).

**Figure 2:**
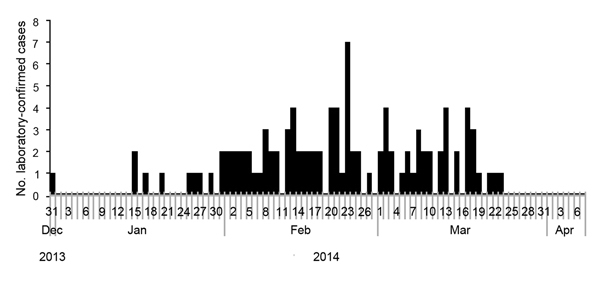
Distribution of 106 laboratory-confirmed Kyasanur Forest disease cases by date of symptom onset, Shimoga District, Karnataka State, India, December 2013–April 2014.

Of the 106 case-patients, 102 were from 41 villages in Thirthahalli Taluk (cumulative population 7,317), and 4 were from 3 villages (cumulative population 559) in Hosanagara Taluk (also in Shimoga District) (Figure 1). The overall attack rate was 13.5 cases/1,000 persons. In affected villages, cases were reported from all age groups; rates were highest among persons >15 years of age and among male residents (Table). Eighteen (16.9%) case-patients were in age groups not targeted for vaccination: 8 were <7 and 10 were >65 years of age. The cases began occurring during the last week of December 2013, peaked during February–March 2014, and then declined gradually (Figure 2).

**Table:** Distribution of patients with laboratory-confirmed Kyasanur Forest disease, by age and sex, Shimoga District, Karnataka State, India, December 31, 2013–April 7, 2014

Characteristic	No. persons infected/no. total in affected villages	Attack rate/1,000 population
Age group, y		
0–4	3/677	4.4
5–14	15/1,347	11.1
15–59	74/5,190	14.3
>60	14/662	21.1
Sex		
M	62/4,001	15.5
F	44/3,875	11.4
Total	106/7,876	13.5

Of the 106 case-patients, 95 (89.6%) reported that they had not been vaccinated and 11 (10.4%) reported being vaccinated (1 received 2 primary and 1 booster dose, 5 received 1 dose, and 5 received 2 doses). Ninety-one case-patients were from villages beyond a 5-km radius of an area with KFD activity in 2013 (i.e., outside an area targeted for vaccination in 2013).

During 2013 (before the outbreak), a total of 19,854 persons 7–65 years of age from Thirthahalli and Hosanagara Taluks had been targeted for KFD vaccination. The coverage of first, second, and booster doses was 23.4%, 15.4%, and 27.3%, respectively.

We included 176 KFD-vaccinated persons (26 received 1 dose, 150 received 2 doses) and 350 unvaccinated persons in the retrospective cohort study. The vaccinated and unvaccinated persons did not differ with respect to age, sex, or occupation (data not shown). Eight laboratory-confirmed KFD case-patients were reported from this cohort (7 were unvaccinated, 1 had received 2 doses of vaccine). The relative risk associated with 1 and 2 doses of vaccine was 0.96 (95% CI 0.06%–16.5%) and 0.33 (95% CI 0.04%–2.69%), respectively. The VE of 1 and 2 doses of vaccine was 4% (0%–96%) and 67% (0%–96%), respectively.

## Conclusions

The findings of our investigation highlighted 4 concerns regarding KFD vaccination strategy practiced in the region. First, vaccine coverage in villages selected for vaccination in 2013 was low. Earlier studies also have shown that nearly half of the eligible population in the targeted villages was not vaccinated (*4*). These findings indicate low acceptance of KFD vaccine, possibly because of vaccine-associated adverse effects and the need for multiple doses. Second, the observed VE was lower than that reported in a previous study (1 dose, 79%; 2 doses, 94%) (*7*). However, other recent reports also indicate lower VE (*2*,*4*,*8*). Third, the occurrence of cases in areas >5 km away from villages vaccinated in the previous year suggests that targeting vaccination to areas within a 5-km radius of reported KFD activity may not be effective in preventing KFDV transmission outside the vaccinated areas. KFDV is primarily transmitted by the bite of infected ticks, and it is spread by the movement of monkeys that carry infected ticks; thus, vaccinating around zones with reported KFD activity is unlikely to prevent spread of the virus. Fourth, ≈17% of the patients in our study with laboratory-confirmed KFDV infection were <7 or >65 years of age, and persons of these ages are not administered KFD vaccine, probably because lower attack rates were observed among these age groups in earlier outbreaks (*7*,*9*,*10*).

Our study had 2 limitations. First, the facility-based surveillance relied on passive detection of case-patients seeking care from the selected health facilities. The number of case-patients detected is influenced by the health-seeking behavior of the community and by the severity of illness. Second, although VE was found to be low, the cohort study had low power (22.3%); hence, the findings of low effectiveness must be interpreted with caution.

To control KFDV, systematic efforts are needed to improve the current vaccine and vaccine coverage. Current vaccination strategies should be reviewed and the reasons for low VE should be evaluated.
